# Endoscopic Ultrasound-Guided Gastroenterostomy for the Palliation of Gastric Outlet Obstruction (GOO): A Systematic Review and Meta-analysis of the Different Techniques

**DOI:** 10.7759/cureus.31526

**Published:** 2022-11-15

**Authors:** Pedro Henrique Boraschi V Ribas, Diogo Turiani H De Moura, Igor M Proença, Epifânio S Do Monte Júnior, Erika Y Yvamoto, Matheus C Hemerly, Victor L De Oliveira, Igor B Ribeiro, Sergio A Sánchez-Luna, Wanderley M Bernardo, Eduardo Guimarães H De Moura

**Affiliations:** 1 Gastroenterology, Hospital das Clínicas HCFMUSP Faculdade de Medicina, Universidade de São Paulo, São Paulo, BRA; 2 Gastroenterology, University of Alabama at Birmingham Marnix E. Heersink School of Medicine, Birmingham, USA

**Keywords:** lumen apposing metal stent, palliation, palliative care, endoscopic ultrasound (eus), lams, eus-ge, goo, gastric outlet obstruction

## Abstract

Introduction: Gastric outlet obstruction (GOO) is usually associated with a poor prognosis and a significant decrease in a patient’s quality of life. Endoscopic ultrasound-guided gastroenterostomy (EUS-GE) using lumen-apposing metal stents (LAMS) has emerged as a safe and effective palliation procedure for GOO in patients that are unfit for surgery. Without an exclusive gold-standard technique for EUS-GE, we aimed to compare the currently available ones in this systematic review and meta-analysis, the first on this subspecialty.

Methods: A comprehensive search from multiple electronic databases was performed. The search had a particular emphasis on the techniques used in performing EUS-GE. We identified all the studies in which EUS-GE was performed as palliation for GOO from its inception to the current date. The outcomes analyzed were the following: technical and clinical success, total and severe adverse events (AEs), procedure duration, and length of hospital stay (LOHS).

Results: Twenty studies involving 863 patients were the basis of this statistical analysis. Patients underwent the following techniques: direct gastroenterostomy (DGE) (n=718), balloon-assisted gastroenterostomy (BAGE) (n=27), and endoscopic ultrasound (EUS)-guided double-balloon-occluded gastrojejunostomy bypass (n=118). In comparison to balloon-assisted techniques, DGE had a lower rate of AEs, -0.121 (95% CI -0.191 to -0.051 p=0.001); and LOHS for the DGE group, -2.684 (95% CI -1.031 to -4.337 p=0.001). The other analyzed outcomes presented no statistically significant differences. On a sub-analysis, BAGE showed a lower rate of AEs than EUS-guided double-balloon-occluded gastrojejunostomy bypass, -0.196 (95% CI -0.061 to -0.331 p=0.004).

Conclusions: EUS-GE is a safe and effective procedure for palliating GOO. When correctly administered, any of the analyzed techniques may be used to palliate GOO with similar technical and clinical outcomes. DGE had significantly lower rates of AEs and LOHS, which can be inferred as a safer procedure. These results should be interpreted cautiously due to the limited few studies that are available and accessible. Therefore, further well-designed, randomized clinical studies on the topic are warranted to compare the different techniques from more sources.

## Introduction and background

Gastric outlet obstruction (GOO) is a potential complication caused by various malignant and benign diseases of the upper gastrointestinal tract, which results in poor emptying of stomach content. When related to malignancy, it is usually associated with a poor prognosis, a decrease in quality of life, and an increase in morbidity. The primary etiologies of malignant GOO include gastric/duodenal cancer, cholangiocarcinoma, lymphomas, and metastasis. The patients' usual signs and symptoms are nausea, vomiting, weight loss, abdominal pain, inability to eat, and ascites [1-2]. 

The palliation approach results are variable and depend on the patient’s clinical status [3-4]. Surgical gastrojejunostomy (SGJ), performed either as an open surgery or laparoscopy, is still preferable in patients with longer life expectancies due to the low reintervention rates. However, it is associated with considerable procedural-related morbidity [3-6]. The endoscopic approach to GOO has emerged with enteral, uncovered, self-expandable metal stents (SEMS). The use of SEMS rose due to their effectiveness and safety profile, especially in those patients with a medium-to-low life expectancy (<3 months). However, tumor ingrowth and the loss of patency of the SEMS happen due to its uncovered nature. Therefore, they are usually associated with the recurrence of symptoms, especially in long-term use (>3 months) [3-5, 7-8]. 

The evolution of endoscopic ultrasound (EUS) has allowed endoscopic ultrasound-guided gastroenterostomy (EUS-GE) that uses lumen-apposing metal stents (LAMS) to emerge as a potential minimally invasive approach. It employs a cautery-enhanced LAMS to bypass the obstruction by creating an anastomosis between the stomach and the jejunum, distal to the obstruction [9-10]. Based on the initial studies done [11-12], it has been proven as a long-lasting luminal patency solution, with minimal risk of tumor ingrowth, surgical risk reduction, shorter procedural time, briefer hospital stays, and fewer adverse events (AEs) [11-12]. 

As a newly developed procedure, some variations have been reported for EUS-GE to achieve jejunal access. The three primary techniques described include direct gastroenterostomy (DGE), balloon-assisted gastroenterostomy (BAGE), and EUS-guided double-balloon-occluded gastrojejunostomy (EPASS) [9, 13]. There is still no absolute and standard technique and only one retrospective comparative study [14] that measured the differences in the approaches. To the best of our knowledge, this is the first systematic review and meta-analysis that evaluate the efficacy and safety of the different techniques for EUS-GE. 

## Review

Materials and methods 

Protocol and Registration 

The study protocol was registered in the International Prospective Register of Systematic Reviews (PROSPERO) under CRD42021272943, approved by the Ethics Committee of Hospital das Clínicas, Faculty of Medicine at The University of São Paulo. This study was performed per the recommendations from the Cochrane Handbook of Systematic Reviews of Interventions and the Preferred Reporting Items for Systematic Reviews and Meta-analysis (PRISMA) guidelines [15]. 

Eligibility Criteria 

All study designs were eligible to be included in this systematic review. Relevant published abstracts and full-text manuscripts describing EUS-GE techniques, regardless of either year of publication or language, were included. All included studies had to provide technical and clinical success and AE rates. Only the most recent study was included when articles concerning sample duplication were identified. The lead author attempted contact to acquire additional data whenever necessary. Studies with missing data and failed contact attempts were excluded. 

Literature Search 

From the study’s inception through October 20, 2022, searches were performed in the following databases based on a standardized protocol. The MEDLINE search strategy was “(gastroenterostomy OR gastroenterostomies OR gastrojejunostomy OR gastrojejunostomies OR Billroth) AND (endoscopy OR endoscopic OR ultrasound OR EUS OR ultrasonography).” An equivalent strategy was performed for EMBASE, Cochrane, Lilacs, and Reference Citation Analysis. Two researchers independently conducted the eligibility screening. Duplicates were excluded, and potential eligible studies were selected for further evaluation. Any disagreements were resolved by consulting a third reviewer. 

Definition of Techniques (Based on the Description of the Included Studies) 

Direct gastroenterostomy (DGE): An endoscopy is performed to fill the duodenum or jejunal loop distal to the obstruction with a mixture of saline, contrast media, and methylene blue. Distal loop distention can be achieved by placing a nasobiliary tube or another catheter that can traverse the obstruction site. The gastric puncture is done either with an ultrasound-guided jejunal loop puncture with a 19-gauge needle followed by an over-the-wire placement of the non-cautery-enhanced LAMS or directly with a cautery-enhanced LAMS (also described as a “freehand” technique). We considered DGE in all the studies that had no balloon assistance for the jejunal/duodenal loop puncture. 

Balloon-assisted gastroenterostomy (BAGE): An endoscopy is performed to place a guidewire and position a dilating balloon through the obstruction in the duodenal or jejunal loop. The balloon is filled with contrast and methylene blue and is punctured with a 19-gauge needle to confirm the correct location. A guidewire can be advanced through the needle, and then a cautery-enhanced LAMS is deployed over the wire. 

Endoscopic ultrasonography-guided balloon occluded gastroenterostomy bypass (EPASS): An endoscopy is performed to place a guidewire or a procedure where an enteroscope with an overtube is placed through the obstruction. After removing the enteroscope or gastroscope, a double-balloon catheter is inserted over the wire or through the overtube. Both balloons are inflated. The fixed segment is filled with contrast and methylene blue. The puncture is performed either directly with cautery-enhanced LAMS or with a 19-gauge needle to confirm the correct location, followed by the guidewire through the needle and the placement of cautery-enhanced LAMS. 

Balloon-assisted techniques (BTGE): The balloon-assisted techniques (EPASS and BAGE) were grouped. 

Data Items and Outcomes Definition 

The selected studies included in the review and meta-analysis had the information extracted based on characteristics of study participants (age, sex, follow-up, primary disease); intervention performed (DGE, BAGE, or EPASS), and outcomes (technical and clinical success, total adverse events (TAEs); and severe adverse events (SAEs), procedure duration, and length of hospital stay (LOHS). 

Technical success was based on the previously published literature. It was defined as “the ability to perform and complete the index procedure” (puncture of the distal bowel, release of the distal flange downstream from the obstruction, and the proximal flange upstream from the obstruction). Clinical success was variable among the studies, although the authors defined as “consuming at least a complete liquid diet without vomiting.” 

Other relevant outcomes were AEs related to the procedure graded according to the lexicon classification for endoscopic AEs set by the 2010 American Society for Gastrointestinal Endoscopy (ASGE) [16]. If the AEs were presented in another classification, they were converted into the previously stated classification by consensus between the researchers and the disputes settled by a third researcher. Procedure duration and LOHS were also extracted. 

The primary analysis was to compare the DGE and the balloon-assisted techniques (BTGE). A subgroup analysis was performed to compare BAGE and EPASS techniques.

Risk of Bias and Quality of Evidence 

The risk of bias was assessed by the Joanna Briggs Institute (JBI) critical appraisal tools, a device for bias evaluation in case series [17], and by Cochrane’s Risk of Bias in Non-randomized Studies of Interventions (ROBINS-I) for the comparative studies [18]. The quality of the evidence was assessed using the objective criteria of the Grading of Recommendations Assessment, Development, and Evaluation (GRADE) for each outcome using the GRADEpro, a guideline development tool software [19]. 

Statistical Analysis 

Comprehensive meta-analysis (CMA) V3 was used to create the tabular and graphical displays and perform the statistical analysis. Absolute values, means, and standard deviations were used in the data analysis. If a study provided medians and measures of variance, mathematical formulae were used to estimate means and standard deviations, thus promoting data standardization, according to Wan et al. [20]. 

The risk difference (RD) was calculated using the Mantel-Haenszel formula for dichotomous variables, with a corresponding confidence interval (CI) of 95%. The difference of means (MD) for continuous variables was calculated with inverse variance and a CI of 95%. All calculated p-values were two-sided, and p-values <0.05 were considered statistically significant. 

Heterogeneity (inconsistency) was assessed and quantified according to the Higgins method (I2). If the heterogeneity (I2) value was greater than 50%, it was considered high, and a random-effects model was chosen to evaluate this data. A fixed-effects model was preferred for the heterogeneity values lower than 50% [21]. A funnel plot was created and visually inspected for asymmetry and quantitative accuracy for publication bias analysis using Egger’s regression testing [22]. 

Results 

We identified 15,730 articles in total. We excluded the articles from the same population in a more extensive, newer study. We also excluded those that did not specify the techniques used or if there were mixed EUS-GE techniques in the pool of patients. A total of 20 studies fulfilled our inclusion criteria: 12 case series and eight comparative studies [12, 14, 23-40]. 

**Figure 1:**
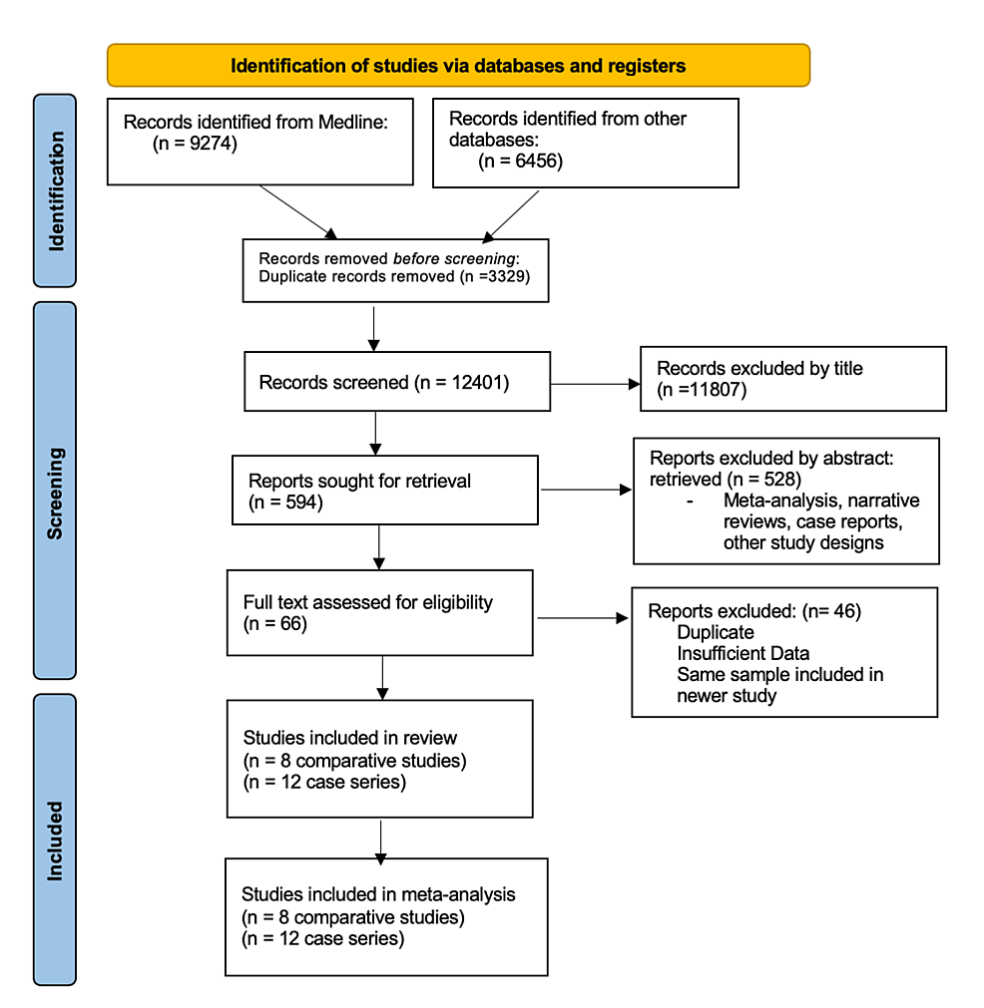
PRISMA flow diagram. PRISMA, Preferred Reporting Items for Systematic Reviews and Meta-analysis

Of those, 15 reported DGE [14, 23-36], and six reported BTGE [12, 14, 37-40]. The total amount of patients was 863-718 in DGE and 145 in BTGE (27 in BAGE and 118 in EPASS), respectively. The PRISMA flow diagram is shown in Figure 1, and individual data from the studies are in Table 1.

**Table 1:** Summary of included studies. DGE, direct gastroenterostomy; BAGE, balloon-assisted gastroenterostomy; EPASS, EUS-guided balloon occluded gastroenterostomy bypass; PC, pancreatic cancer; DC, duodenal cancer; GC, gastric cancer; CC, colorectal cancer; /GC, biliary/gallbladder cancer; BC, breast cancer; MC, metastatic cancer; NET, neuroendocrine tumor; AC, ampullary cancer; D/AC, duodenal/ampullary cancer; P/BC, pancreatic/biliary cancer; BD, benign disease; SB, small bowel cancer; LOHS, length of hospital stay; PD, procedure duration; TS, technical success; CS, clinical success; TAE, total adverse events; SAE: severe adverse events

Included studies	Study type	Technique	No of patients	Age (years ± SD)	Female (%)	Primary disease N (%)	Follow-up (days ± SD)	Outcomes
Abbas et al. (2021) [30]	Retrospective case series	DGE	50	60 ± 10	27 (54%)	PC 25 (50%), B/GC 6 (12%), SB 1 (2%), GC 2 (4%), other 16 (32%)	Not available	TS, CS; TAE; SAE; LOHS; PD
Chen et al. (2018) [14]	Retrospective comparative	DGE	52	62.9 ± 11.3	30 (57.7%)	PC 9 (17.3%), B/GC 1 (1.9%), GC 1 (1.9%), D/AC 9 (17.3%), MC 14 (26.9%), BD 18 (34.6%)	120 ± 26.8	TS, CS; TAE; SAE; LOHS; PD
Chen et al. (2018) [14]	Retrospective comparative	BAGE	22	63.3 ± 12.3	11 (50%)	PC 1 (4.6%), B/GC 2 (9.1%), GC 2 (9.1%), D/AC 1 (4.6%), MC 8 (36.4%), BD 7 (31.8%)	120 ± 26.8	TS, CS; TAE; SAE; LOHS; PD
Fischer et al. (2021) [33]	Retrospective case series	DGE	44	66	26 (59%)	Not available	Not available	TS, CS; TAE; SAE
Sobani et al. (2021) [34]	Retrospective case series	DGE	31	61.3 ± 16.5	14 (45%)	PC 10 (32%), B/GC 4 (13%), DC 4 (13%), GC 1 (3%), MC 4 (13%), BD 8 (26%)	140 ± 160	TS, CS; TAE; SAE
Hu et al. (2020) [24]	Retrospective case series	DGE	10	63.2 ± 5.8	6 (60%)	PC 7 (70%), GC 1 (10%), DC 1 (10%), BD 1 (10%)	Not available	TS, CS; TAE; SAE; PD
Itoi et al. (2016) [12]	Retrospective case series	EPASS	20	68 ± 11	10 (50%)	PC 10 (50%), B/GC 1 (5%), GC 5 (25%), DC/A 2 (10%), MC 2 (10%)	119 ± 47.2	TS, CS; TAE; SAE; PD
Jovani et al. (2021) [25]	Retrospective case series	DGE	73	60 ± 15	37 (50.1%)	P/BC 44 (60%), other cancer 20 (27.4%), BD 9 (12.6%)	86 ± 139	TS, CS; TAE; SAE; LOHS; PD
Kerdsirichairat et al. (2019) [26]	Retrospective case series	DGE	57	65	29 (50.1%)	PC 34 (59.6%), MC 8 (14%), DC/A 4 (7%), B/GC 2 (3.5%), BD 9 (15.8%)	180 ± 57	TS, CS; TAE; SAE; LOHS; PD
Kouanda et al. (2021) [27]	Retrospective cohort	DGE	40	70.5 ± 11.5	17 (42.5%)	PC 26 (72.2%), AC 1 (2.8%), DC 1 (2.8%), B/GC 1 (2.8%), MC 5 (13.9%), BD 4 (10%)	140 ± 194	TS, CS; TAE; SAE; LOHS; PD
Marino et al. (2021) [37]	Retrospective case series	EPASS	11	64.9 ± 8.6	5 (45.4%)	PC 8 (73%), GC 1 (9%), DC 1 (9%), MC 1 (9%)	84	TS, CS; TAE; SAE; PD
Nguyen et al. (2021) [29]	Retrospective case series	DGE	42	73.1	19 (45.2%)	PC 29 (69%), B/GC 1 (2.4%), AC 1 (2.4%), DC 1 (2.4%), MC 4 (9.5%), NET 1 (2.4%), BD 5 (12%)	171 ± 505	TS, CS; TAE; SAE; PD
Park et al. (2022) [25]	Retrospective comparative	DGE	36	70.8	18 (50%)	Not available	Not available	TS, CS; TAE; SAE
Sánchez-Aldehuelo et al. (2022) [36]	Retrospective comparative	DGE	79	72.4 ± 10.7	36 (45.5%)	PC 49 (62%), B/GC 5 (6%), GC 15 (19%), DC 5 (6%), other 5 (6%)	Not available	TS, CS; TAE; SAE
Havre et al. (2021) [28]	Retrospective case series	DGE	33	73 ± 13.3	13 (39.4%)	PC 8 (24%), DC 5 (15%), CC 6 (18%), B/GC 2 (6%), MC 3 (9%), Linfoma 1 (3%), other cancers 3 (9%), BD 5 (15%)	Not available	TS, CS; TAE; SAE; LOHS; PD
Huang et al. (2022) [40]	Retrospective comparative	EPASS	51	65.8 ± 13.8	24 (47%)	PC 15 (29%), B/GC 10 (20%), GC 8 (16%), DC 7 (14%), AC 4 (7%), other 7 (14%)	Not available	TS, CS; TAE; LOHS; PD
Urrehman et al. (2018) [39]	Prospective case series	BAGE	5	64.75 ± 12.7	Not available	PC 4 (80%), DC 1 (20%)	30-180	TS, CS; TAE; SAE; PD
Vazquez-Sequeiros et al. (2020) [32]	Retrospective comparative	DGE	46	72.7 ± 11.2	19 (41.3%)	PC 28 (61%), GC 7 (46%), DC 3 (6%), B/GC 4 (9%), other diseases 4 (9%)	134 ± 110	TS, CS; TAE; SAE
Van Wanrooij et al. (2022) [23]	Retrospective comparative	DGE	88	66 ± 12.1	44 (50%)	PC 50 (57%), B/GC 11 (12%), GC 8 (9%), DC 8 (9%), other 11 (12%)	110 ± 106	TS, CS; TAE; SAE; LOHS
Westerveld et al. (2021) [31]	Retrospective comparative	DGE	37	67.5 ± 12.8	22 (33%)	Not available	Not available	TS, CS; TAE; SAE; PD
Xu et al. (2020) [38]	Retrospective case series	EPASS	36	69 ± 12.8	19 (52.8%)	PC 15 (41.7%), GC 4 (11.1%), B/GC 8 (22.2%), MC 4 (11.1%)	89	TS, CS; TAE; SAE; LOHS; PD

Risk of Bias and Quality of the Evidence

Joanna Briggs Institute Critical Appraisal Tools assessed the risk of bias for the case series (Table 2) and ROBINS-I for comparative studies (Table 3). The quality of evidence for each outcome is described in Tables 4-5.

**Table 2:** Joanna Briggs risk of bias assessment.

Study	Inclusion criteria	Condition evaluation	Condition identification	Consecutive inclusion	Complete inclusion	Study demographic report	Clinical information	Outcomes and follow-up	Site demographic information	Statistical analysis	Overall bias assessment
Abbas et al. (2021) [30]	Yes	Yes	Yes	Yes	Yes	Yes	Yes	Yes	Yes	Yes	Low
Fischer et al. (2021) [33]	Unclear	Unclear	Unclear	Yes	Unclear	No	No	No	No	Yes	High
Havre et al. (2021) [28]	Yes	Yes	Yes	Yes	Yes	Yes	No	Yes	Yes	Yes	Low
Hu et al. (2020) [24]	Yes	Unclear	Yes	Yes	Yes	Yes	No	No	Yes	Yes	Moderate
Itoi et al. (2016) [12]	Yes	Yes	Yes	Yes	Yes	Yes	Yes	Yes	Yes	Yes	Low
Jovani et al. (2021) [25]	Yes	Yes	Yes	Yes	Yes	Yes	Yes	Yes	Yes	Yes	Low
Kerdsirichairat et al. (2019) [26]	Yes	Yes	Yes	Yes	Yes	Yes	Yes	Yes	Yes	Yes	Low
Marino et al. (2021) [37]	Yes	Yes	Yes	Yes	Yes	Yes	Yes	Yes	Yes	Yes	Low
Nguyen et al. (2021) [29]	Yes	Yes	Yes	Yes	Yes	Yes	Yes	Yes	Yes	Yes	Low
Sobani et al. (2021) [34]	Yes	Yes	Yes	Yes	Yes	Yes	Yes	Yes	Yes	Yes	Low
Urrehman et al. (2018) [39]	Yes	Yes	Yes	Unclear	Unclear	No	No	Yes	No	Yes	Moderate
Xu et al. (2020) [38]	Yes	Yes	Yes	Yes	Yes	Yes	Yes	Yes	Yes	Yes	Low

**Table 3:** ROBINS-I risk of bias assessment.

Study	Bias due to confounding	Bias due to the selection of participants	Bias in the classification of interventions	Bias due to deviations from intended interventions	Bias due to missing data	Bias in the measurement of the outcomes	Bias in the selection of reported result	Overall bias assessment
Chen et al. (2018) [14]	Moderate	Moderate	Low	Low	Low	Low	Low	Moderate
Kouanda et al. (2021) [27]	Moderate	Moderate	Low	Low	Low	Low	Low	Moderate
Huang et al. (2022) [40]	Moderate	Moderate	Low	Low	Moderate	Low	Low	Moderate
Park et al. (2022) [25]	Moderate	Moderate	Low	Low	Low	Low	Low	Moderate
Sánchez-Aldehuelo et al. (2022) [36]	Moderate	Moderate	Low	Low	Low	Low	Low	Moderate
Vazquez-Sequeiros et al. (2020) [32]	Moderate	Moderate	Low	Low	No information	Low	Low	Moderate
Van Wanrooij et al. (2022) [23]	Moderate	Moderate	Low	Low	Low	Low	Low	Moderate
Westerveld et al. (2021) [31]	Moderate	Moderate	Low	Low	No information	Low	Low	Moderate

**Table 4:** GRADE quality of evidence, DGE vs. BTGE. BTGE, balloon-assisted techniques gastroenterostomy; DGE, direct gastroenterostomy; CI, confidence interval; RR, risk ratio; LOHS, length of hospital stay; TAEs, adverse events; SAEs, severe adverse event a. Egger’s regression test and Funnel plot showed possible publication bias; b. High heterogeneity; c. The superior confidence interval is higher than two times the median; d. Procedure duration alone does not evaluate the best intervention; e. The LOHS alone does not help to evaluate the best intervention

Certainty assessment							Summary of findings				
Participants (studies) follow-up	Risk of bias	Inconsistency	Indirectness	Imprecision	Publication bias	Overall certainty of the evidence	Study event rates (%)		Relative effect (95% CI)	Anticipated absolute effects	
							With BTGE	With DGE		Risk with BTGE	Risk difference with DGE
Technical Success											
863 (20 observational studies) [12,14,23-40]	Not serious	Not serious	Not serious	Not serious	Publication bias strongly suspected^a^	⨁⨁⨁◯Moderate	136/145 (93.8%)	680/718 (94.7%)	RR 0.987 (0.943-1.034)	938 per 1,000	12 fewer per 1,000 (from 55 fewer to 31 more)
Clinical Success											
863 (20 observational studies) [12,14,23-40]	Not serious	Not serious	Not serious	Not serious	Publication bias strongly suspected^a^	⨁⨁⨁◯Moderate	129/145 (89.0%)	648/718 (90.3%)	RR 0.992 (0.935-1.053)	890 per 1,000	7 fewer per 1,000 (from 6 fewer to 46 more)
TAEs											
863 (20 observational studies) [12,14,23-40]	Not serious	Not serious	Not serious	Not serious	Publication bias strongly suspected^a^	⨁⨁⨁◯Moderate	31/145 (21.4%)	67/718 (9.3%)	RR 0.435 (0.295-0.640)	214 per 1,000	121 fewer per 1,000 (from 191 fewer to 51 fewer)
SAEs											
812 (19 observational studies) [12,14,23-39]	Not serious	Serious^b^	not serious	Serious^c^	Publication bias strongly suspected^a^	⨁◯◯◯Very low	8/94 (8.5%)	24/718 (3.3%)	RR 0.415 (0.190-0.905)	85 per 1,000	48 fewer per 1,000 (from 195 fewer to 9 more)
Procedure Duration											
539 (14 observational studies) [12,14,24-31,37-40]	Not serious	Very serious^b^	Serious^d^	Serious^c^	publication bias strongly suspected^a^	⨁◯◯◯Very low	145	394	-	The mean procedure duration was 64.74 min	Mean 16.26 minutes lower (5.23 lower to 37.75 higher)
LOHS											
513 (10 observational studies) [14,23,25-28,30,37,38,40]	Not serious	Serious^b^	Serious^e^	Not serious	None	⨁⨁◯◯Low	120	393	-	The mean LOHS was 6.85 days	Mean 2.684 days lower (1,031 lower to 4,337 lower)

﻿

**Table 5:** GRADE quality of evidence BAGE vs. EPASS. BAGE, balloon-assisted gastroenterostomy; EPASS, EUS-guided double-balloon-occluded gastrojejunostomy bypass; CI: confidence interval; MD: mean difference; RR: risk ratio; LOHS, length of hospital stay; TAE, total adverse events; SAE, severe adverse events a. High heterogeneity; b. The superior confidence interval is higher than two times the median; c. Egger’s regression test and Funnel plot showed possible publication bias; d. Procedure duration alone does not evaluate the best intervention; e. The LOHS alone does not help to evaluate the best intervention

Certainty assessment							Summary of findings				
Participants (studies) follow-up	Risk of bias	Inconsistency	Indirectness	Imprecision	Publication bias	Overall certainty of the evidence	Study event rates (%)		Relative effect (95% CI)	Anticipated absolute effects	
							With EPASS	With BAGE		Risk with EPASS	Risk difference with BAGE
Technical Success											
145 (6 observational studies) [12,14,37-40]	Not serious	Not serious	Not serious	Not serious	None	⨁⨁⨁⨁High	111/118 (94.1%)	24/27 (88.9%)	RR 0.963 (0.849-1.092)	941 per 1,000	35 fewer per 1,000 (from 150 fewer to 80 more)
Clinical Success											
145 (6 observational studies) [12, 14, 37-40]	Not serious	Not serious	not serious	not serious	none	⨁⨁⨁⨁High	105/118 (89.0%)	24/27 (88.9%)	RR 1.018 (0.891-1.163)	890 per 1,000	16 more per 1,000 (from 105 fewer to 137 more)
TAEs											
145 (6 observational studies) [12, 14, 37-40]	Not serious	Serious^a^	Not serious	Serious^b^	Publication bias strongly suspected^c^	⨁◯◯◯Very low	33/118 (28.0%)	2/27 (7.4%)	RR 3.202 (0.927-11.068)	280 per 1,000	106 more per 1,000 (from 61 more to 331 more)
SAEs											
94 (5 observational studies) [12, 14, 37-39]	Not serious	Not serious	Not serious	Serious^b^	None	⨁⨁⨁◯Moderate	6/67 (9.0%)	1/27 (3.7%)	RR 0.452 (0.064-3.176)	90 per 1,000	51 fewer per 1,000 (from 154 fewer to 52 more)
Procedure Duration											
145 (6 observational studies) [12, 14, 37-40]	Not serious	Very serious^a^	Serious^d^	Serious^b^	Publication bias strongly suspected^c^	⨁◯◯◯Very low	118	27	-	The mean procedure duration was 54.07 min	MD 35.8 min higher (18.83 lower to 90.53 higher)
LOHS											
120 (4 observational studies) [14, 37-38, 40]	Not serious	Serious^a^	Serious^e^	Very serious^b^	None	⨁◯◯◯Very low	98	22	-	The mean LOHS was 7.3 days	MD 1.83 days lower (6.17 lower to 2.21 higher)

Meta-analysis

DGE Versus BTGE

Technical Success

**Figure 2:**
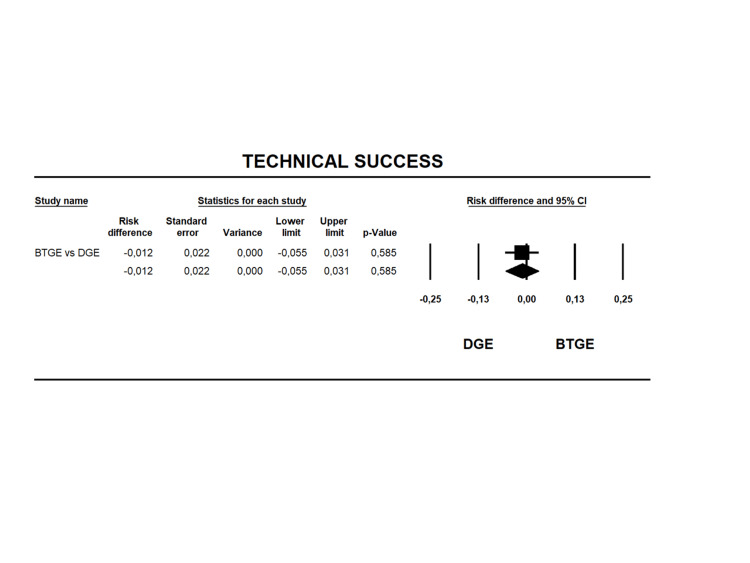
Forest plot for risk difference in technical success between BTGE and DGE. DGE, direct gastroenterostomy; BTGE, balloon-assisted techniques gastroenterostomy

All studies [12, 14, 23-40] were included in this analysis. The technical success was 94.8% in the direct puncture group and 93.6% in the balloon-assisted group (Table 6), with an RD of -0.012 (95% CI -0.055 to 0.031 I2= 0% p=0.585) without statistical difference between the groups (Figure 2). The quality of evidence for this outcome was moderate (Table 4).

**Table 6:** Summary of DGE and BTGE outcomes. DGE, direct gastroenterostomy; BTGE, balloon-assisted techniques gastroenterostomy; CI, confidence interval; SD, standard deviation; TAE, total adverse events; SAE, severe adverse events; LOHS, length of hospital stay

Outcomes	Included studies in meta-analysis	Event rate (lower and upper limit)	Mean ± SD	p Value (CI 95%)
DGE				
Technical success	Abbas et al. [30]/Chen et al. [14]/Fischer et al. [33]/Havre et al. [28]/Hu et al. [24]/Jovani et al. [25]/Kerdsirichairat et al. [26]/Kouanda et al. [27]/Nguyen et al. [29]/Park et al. [35]/Sanchez-Aldehuelo et al. [36]/Sobani et al. [34]/Van Wanrooij et al. [23]/Vazquez-Sequeiros et al. [32]/Westerveld et al. [31]	0.948 (0.928–0.963)	N/A	0
Clinical success	Abbas et al. [30]/Chen et al. [14]/Fischer et al. [33]/Havre et al. [28]/Hu et al. [24]/Jovani et al. [25]/Kerdsirichairat et al. [26]/Kouanda et al. [27]/Nguyen et al. [29]/Park et al. [35]/Sanchez-Aldehuelo et al. [36]/Sobani et al. [34]/Van Wanrooij et al. [23]/Vazquez-Sequeiros et al. [32]/Westerveld et al. [31]	0.906 (0.882–0.925)	N/A	0
TAE	Abbas et al. [30]/Chen et al. [14]/Fischer et al. [33]/Havre et al. [28]/Hu et al. [24]/Jovani et al. [25]/Kerdsirichairat et al. [26]/Kouanda et al. [27]/Nguyen et al. [29]/Park et al. [35]/Sanchez-Aldehuelo et al. [36]/Sobani et al. [34]/Van Wanrooij et al. [23]/Vazquez-Sequeiros et al. [32]/Westerveld et al. [31]	0.093 (0.063–0.135)	N/A	0
SAE	Abbas et al. [30]/Chen et al. [14]/Fischer et al. [33]/Havre et al. [28]/Hu et al. [24]/Jovani et al. [25]/Kerdsirichairat et al. [26]/Kouanda et al. [27]/Nguyen et al. [29]/Park et al. [35]/Sanchez-Aldehuelo et al. [36]/Sobani et al. [34]/Van Wanrooij et al. [23]/Vazquez-Sequeiros et al. [32]/Westerveld et al. [31]	0.034 (0.022–0.053)	N/A	0
Procedure duration	Abbas et al. [30]/Chen et al. [14]/Havre et al. [28]/Hu et al. [24]/Jovani et al. [25]/Kerdsirichairat et al. [26]/Kouanda et al. [27]/Nguyen et al. [29]/Westerveld et al. [31]	N/A	64.74 ± 153.6 min	0
LOHS	Abbas et al. [30]/Chen et al. [14]/Havre et al. [28]/Jovani et al. [25]/Kerdsirichairat et al. [26]/Kouanda et al. [27]/Van Wanrooij et al. [23]	N/A	4.17 ± 7.1 days	0
BTGE				
Technical success	Chen et al. [14]/Urrehman et al. [39]/Itoi et al. [12]/Huang et al. [40]/Marino et al. [37]/Xu et al. [38]	0.936 (0.870–0.969)	N/A	0
Clinical success	Chen et al. [14]/Urrehman et al. [39]/Itoi et al. [12]/Huang et al. [40]/Marino et al. [37]/Xu et al. [38]	0.899 (0.834–0.940)	N/A	0
TAE	Chen et al. [14]/Urrehman et al. [39]/Itoi et al. [12]/Huang et al. [40]/Marino et al. [37]/Xu et al. [38]	0.214 (0.091–0.423)	N/A	0.01
SAE	Chen et al. [14]/Urrehman et al. [39]/Itoi et al. [12]/Marino et al. [37]/Xu et al. [38]	0.082 (0.038–0.167)	N/A	0
Procedure duration	Chen et al. [14]/Urrehman et al. [39]/Itoi et al. [12]/Huang et al. [40]/Marino et al. [37]/Xu et al. [38]	N/A	48.21 ± 93.29 min	0
LOHS	Chen et al. [14]/Huang et al. [40]/Marino et al. [37]/Xu et al. [38]	N/A	6.85 ± 9.33 days	0

Clinical Success

**Figure 3:**
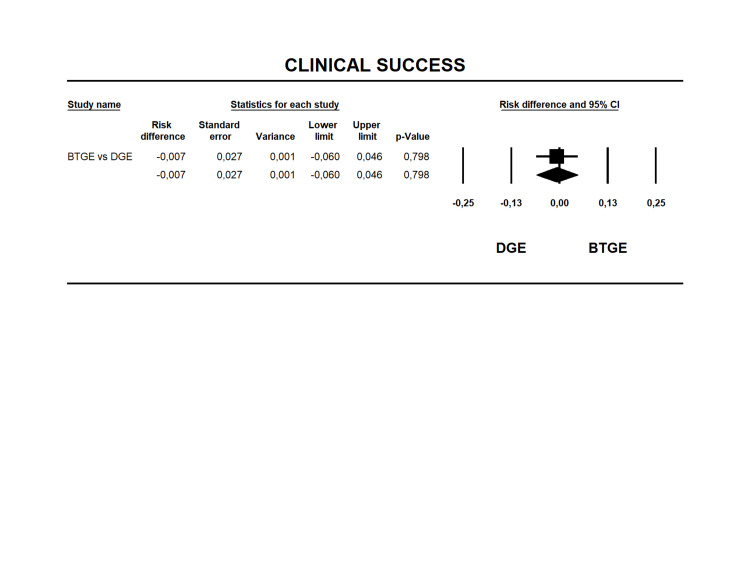
Forest plot for risk difference in clinical success between BTGE and DGE. DGE, direct gastroenterostomy; BTGE, balloon-assisted techniques gastroenterostomy

All studies reported clinical success [12, 14, 23-40]. Clinical success rates were 90.6% for DGE and 88.9% for BTGE (Table 6) with an RD of -0.007 (95% CI -0.06 to 0.046 I2=0% p=0.798) without statistical difference between the groups (Figure 3). The quality of evidence for this outcome was moderate (Table 4).

Total Adverse Events

**Figure 4:**
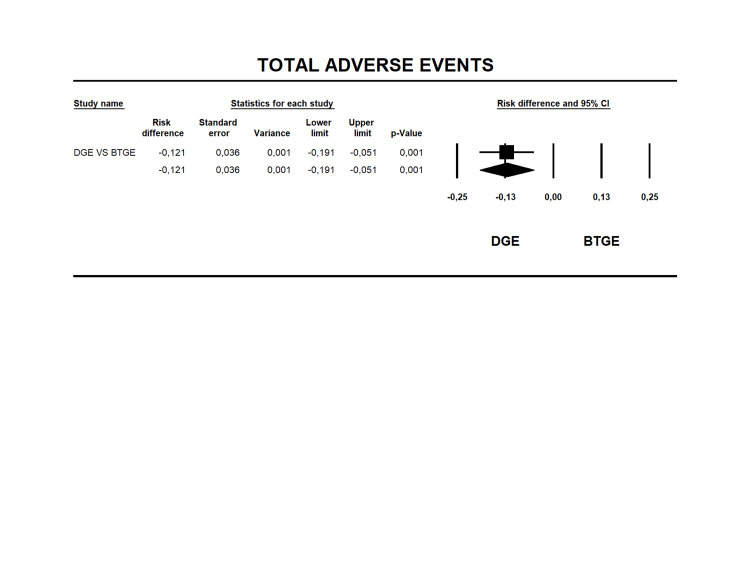
Forest plot for risk difference in TAEs between BTGE and DGE. DGE, direct gastroenterostomy; BTGE, balloon-assisted techniques gastroenterostomy; TAEs, total adverse events

All studies reported AEs [12, 14, 23-40]. The rates were 9.3% and 21.4% for DGE and BTGE (Table 6), respectively. RD was -0.121 (95% CI -0.191 to -0.051 I2=77.1% p=0.001), showing a lower risk of AEs on the DGE (Figure 4). The quality of evidence for this outcome was moderate (Table 4).

Severe Adverse Events

**Figure 5:**
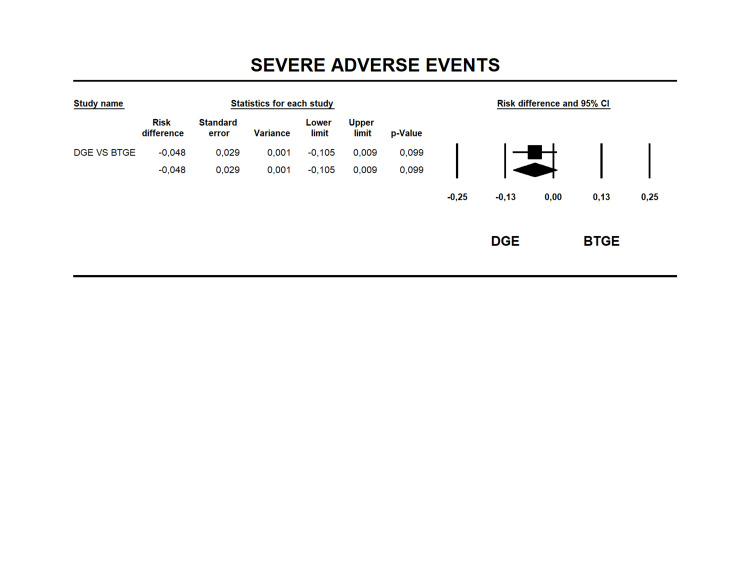
Forest plot for risk difference for SAEs between BTGE and DGE. DGE, direct gastroenterostomy; BTGE, balloon-assisted techniques gastroenterostomy; SAEs, severe adverse events

Nineteen studies reported severe AEs [12, 14, 23-39]. The rate was 3.4% and 8.2% for DGE and BTGE (Table 6), respectively. The RD was -0.048 (95% CI -0.105 to 0.009 I2=0% p=0.099), without a statistical difference between the groups (Figure 5). The quality of evidence for this outcome was very low (Table 4).

Procedure Duration

**Figure 6:**
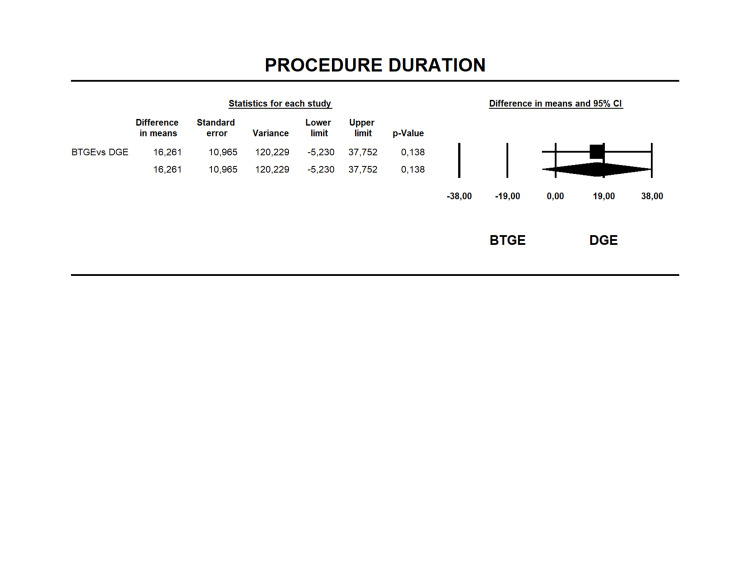
Forest plot for difference in means in procedure duration for BTGE and DGE. DGE, direct gastroenterostomy; BTGE, balloon-assisted techniques gastroenterostomy

Fourteen studies [12, 14, 24-31, 37-40] reported procedure duration. The mean duration was 48.21±93.29 min and 64.74±153.6 min for DGE and BTGE (Table 6), respectively. MD was 16.26 min (95% CI -5.23 to 37.75 I2=97% p=0.138) without a statistical difference (Figure 6). The quality of evidence for this outcome was very low (Table 4).

Length of Hospital Stay

**Figure 7:**
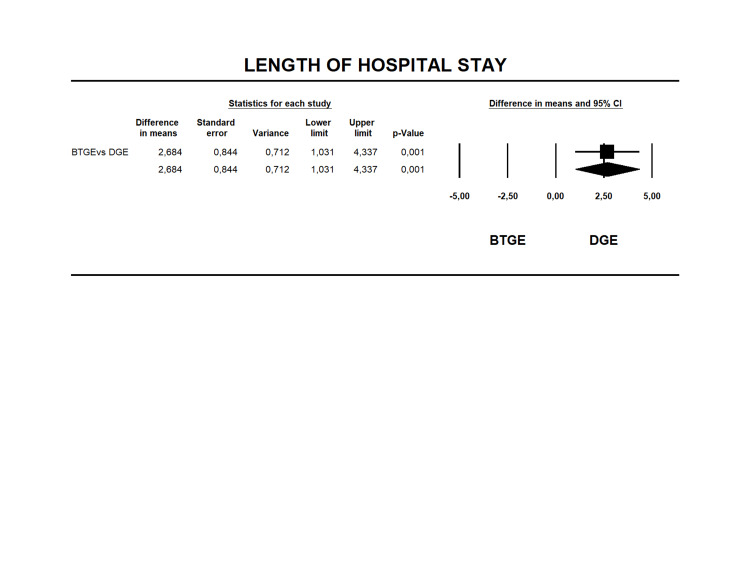
Forest plot for difference in means in LOHS for BTGE and DGE. DGE, direct gastroenterostomy; BTGE, balloon-assisted techniques gastroenterostomy; LOHS, length of hospital stay

Ten studies [14, 23, 25-28, 30, 37-38, 40] reported the LOHS. The hospital stay was 4.17 ± 7.1 days and 6.85 ± 9.33 days (Table 6), respectively. The MD was 2.684 (95% CI 1.031-4.337 I2=68% p=0.001), with a shorter hospitalization period in the DGE group (Figure 7). The quality of evidence for this outcome was low (Table 4).

EPASS Versus BAGE

Technical Success

Six studies [12, 14, 37-40] reported technical success in this subgroup analysis. The BAGE group had 91.1%, and the EPASS group had 94.6% technical success with an RD of -0.035 (95% CI -0.150 to 0.080 I2=5% p=0.550), without statistical significance between the groups (Table 7). The quality of evidence for this outcome was high (Table 5).

**Table 7:** Summary of BAGE vs. EPASS outcomes. BAGE, balloon-assisted gastroenterostomy; EPASS, EUS-guided double-balloon-occluded gastrojejunostomy bypass; TAE, total adverse events; SAE, severe adverse events; LOHS, length of hospital stay

Outcomes	Included studies in meta-analysis	Event rate (lower and upper limits)	Mean ± SD	Risk difference (lower and upper limits)	Difference in means (lower and upper limits)	p value (CI 95%)
BAGE						
Technical success	Chen et al. [14]/Urrehman et al. [39]	0.911 (0.736–0.974)	N/A	N/A	N/A	0
Clinical success	Chen et al. [14]/Urrehman et al. [39]	0.911 (0.736–0.974)	N/A	N/A	N/A	0
AE	Chen et al. [14]/Urrehman et al. [39]	0.089 (0.026–0.264)	N/A	N/A	N/A	0
SAE	Chen et al. [14]/Urrehman et al. [39]	0.042 (0.006–0.248)	N/A	N/A	N/A	0.002
Procedure duration	Chen et al. [14]/Urrehman et al. [39]	N/A	89.9 ± 35.3 min	N/A	N/A	0
LOHS	Chen et al. [14]	N/A	5.5 ± 5 days	N/A	N/A	0
EPASS						
Technical success	Itoi et al. [12]/Huang et al. [40]/Marino et al. [37]/Xu et al. [38]	0.946 (0.871–0.979)	N/A	N/A	N/A	0
Clinical success	Itoi et al. [12]/Huang et al. [40]/Marino et al. [37]/Xu et al. [38]	0.895 (0.820–0.942)	N/A	N/A	N/A	0
AE	Itoi et al. [12]/Huang et al. [40]/Marino et al. [37]/Xu et al. [38]	0.285 (0.123–0.530)	N/A	N/A	N/A	0.083
SAE	Itoi et al. [12]/Marino et al. [37]/Xu et al. [38]	0.093 (0.041–0.198)	N/A	N/A	N/A	0
Procedure duration	Itoi et al. [12]/Huang et al. [40]/Marino et al. [37]/Xu et al. [38]	N/A	54.1 ± 143.02 min	N/A	N/A	0
LOHS	Huang et al. [40]/Marino et al. [37]/Xu et al. [38]	N/A	7.3 ± 10 days	N/A	N/A	0
BAGE vs EPASS						
Technical success	Chen et al. [14]/Urrehman et al. [39]/Itoi et al. [12]/Huang et al. [40]/Marino et al. [37]/Xu et al. [38]	N/A	N/A	-0.035 (-0.150–0.080)	N/A	0.550
Clinical success	Chen et al. [14]/Urrehman et al. [39]/Itoi et al. [12]/Huang et al. [40]/Marino et al. [37]/Xu et al. [38]	N/A	N/A	0.016 (-0.103–0.137)	N/A	0.795
TAE	Chen et al. [14]/Urrehman et al. [39]/Itoi et al. [12]/Huang et al. [40]/Marino et al. [37]/Xu et al. [38]	N/A	N/A	-0.196 (-0.061– -0.311)	N/A	0.004
SAE	Chen et al. [14]/Urrehman et al. [39]/Itoi et al. [12]/Marino et al. [37]/Xu et al. [38]	N/A	N/A	-0.051 (-0.154–0.052)	N/A	0.331
Procedure duration	Chen et al. [14]/Urrehman et al. [39]/Itoi et al. [12]/Huang et al. [40]/Marino et al. [37]/Xu et al. [38]	N/A	N/A	N/A	35.8 min (-18.8–90.5)	0.199
LOHS	Chen et al. [14]/Huang et al. [40]/Marino et al. [37]/Xu et al. [38]	N/A	N/A	N/A	-1.83 days (-2.21–6.17)	0.409

Clinical Success

Six studies [12, 14, 37-40] reported clinical success in this subgroup analysis. BAGE had 91.1% of clinical success while EPASS had 89.5%, with an RD of 0.016 (95% CI -0.105 to 0.137 I2=29% p=0.795), without a statistical difference between the groups (Table 7). The quality of evidence for this outcome was high (Table 5).

Total Adverse Events

Six studies [12, 14, 37-40] reported AEs in this subgroup analysis,. BAGE had 8.9% of total AEs while EPASS had 28.5%, with an RD of 0.196 (95% CI 0.061 to 0.331 I2=78% p=0.004), with a lower risk of AEs on the BAGE group (Table 7). The quality of evidence for this outcome was very low (Table 5). 

Severe Adverse Events

Five studies [12, 14, 37-39] reported SAEs in this subgroup analysis. BAGE had 4.2% of severe AEs while EPASS had 9.3%, with an RD of -0.051 (95% CI -0.154 to 0.052 I2=0% p=0.331), without a statistical significance (Table 7). The quality of evidence for this outcome was moderate (Table 5). 

Procedure Duration

Six studies [12, 14, 37-40] reported procedure duration in this subgroup analysis. BAGE had 89.93 ± 35.33 min of procedure duration while EPASS had 54.07±143.02 min, with an MD of 35.8 (95% CI -18.83 to 90.53 I2=97% p=0.199), without statistical significance between the groups (Table 7). The quality of evidence for this outcome was very low (Table 5). 

Length of Hospital Stay

Four studies [14, 37-38, 40] reported LOHS in this subgroup analysis. BAGE had 5.5 ± 5 days of hospital stay while EPASS had 7.3 ± 10 days, with an MD of 1.83 (95% CI -2.21 to 6.17 I2=69% p=0.409), without statistical significance between the groups (Table 7). The quality of evidence for this outcome was very low (Table 5).

Discussion

To our knowledge, no other systematic review with meta-analysis comparing EUS-GE techniques is on record. However, previous reviews have provided comparisons of EUS-GE with surgical gastroenterostomy (SGJ) and enteral stenting (ES). 

Boghossian et al. [41] recently published a systematic review with meta-analysis comparing EUS-GE with ES and SGJ. SGJ is a well-established treatment and is still the benchmark for patients with longer life expectancies (>3 months). It demands a good clinical status but has a low reintervention rate of 10% and high technical and clinical success (100% and 90%, respectively). ES is a good option for patients with a short life expectancies (<3 months); it has a high technical success of 98%, an early diet acceptance, and requires only a short LOHS (1.4 days shorter than EUS-GE) [41]. Compared to EUS-GE, ES has a higher reintervention rate (28% vs. 6%) and severe AE rate (31% vs. 11%), with similar technical success. SGJ has higher technical success than EUS-GE (100% vs. 91%), with similar clinical success (90% vs. 86%) and AE rate (11% vs. 10%) but has an extended LOHS (5 days longer than EUS-GE). Each patient must have an individualized approach, but both these reviews demonstrate that EUS-GE has the potential to become the exclusive standard for most cases [41].

Out of all the outcomes analyzed in our systematic review and meta-analysis, the TAEs and LOHS showed a statistically significant reduction in DGE compared to BTGE. Previous studies [12, 42] have presented the hypothesis that the balloon-assisted techniques were safer and had lower AEs. DGE could have a possible disadvantage considering that an unassisted procedure (without a balloon catheter) may have an increased risk of an inadvertent puncture of a distal bowel loop or colon (as their differentiation under fluoroscopy may be tricky). 

That was proven to be nonidentical from our study, where the incidence of AEs was significantly higher in the BTGE group than in the DGE group, with 21.4% and 9.3%, respectively. With the usage of a catheter to trespass the obstruction and the instillation of saline fluid combined with methylene blue to dilate the small bowel loop, the “freehand” puncture (DGE) is confirmed to be a suitable method. Using a guidewire through the puncture needle can push the loop away, increasing the risk of AEs, as some of the included studies [14, 30] have suggested. The rates for SAEs were 3.1% for DGE and 8.2% for BTGE without a statistically significant difference. Caution should be taken when interpreting the lack of statistical significance in severe AEs, as the rate for DGE is 62% lower. The low number of patients and studies in the BTGE group may interfere with the lack of significance of these findings.

The hospitalization period alone could not allow us to conclude that any technique is superior to the others since different hospitals and centers have different discharge protocols. With lower AE rates in DGE, their association allows us to consider that this technique is safer for these patients, directly affecting their quality of life. 

Although DGE and BTGE had similar technical success, the former has the advantage since it is less laborious and requires fewer materials to perform a functional gastroenterostomy compared to the balloon-assisted techniques. Using fewer materials may decrease the total procedure cost and the learning curve. However, we could not evaluate this due to the scarcity of data available in the included studies. In general, there are fewer studies with a lower number of total procedures about the balloon-assisted techniques in which they are exclusively performed. Their main advantages are the visualization of the balloon(s) and creating a “safe window” of the fixated small bowel, which may facilitate correctly puncturing the desired jejunal loop. One of the disadvantages for the BTGE group is the unavailability of essential materials, such as the double-balloon catheter used in EPASS. This catheter has been developed specifically for this procedure, but it is available mainly in Asia, limiting its adoption in Europe and America. All techniques included in this study can be performed using a guidewire through the 19-gauge puncture before the insertion of the LAMS. Nevertheless, this technique, in contrast to the “freehand” technique, has been associated with a higher rate of stent misdeployments, decreased technical success rates, and increased AEs [42-43] due to the unintended pushing of the jejunal loop before LAMS deployment.

The clinical success was subjectively assessed and mainly from previous patient report charts and records, as most of the included studies are retrospective. The oral intake that varied from liquid to a complete diet was reported as clinically successful. Therefore, a vast difference in the quality of life is portrayed within the same group. This outcome needs to be assessed with more objective parameters in future studies.

Different from our study, Chen et al. [14] conducted the only comparative EUS-GE on technique study that demonstrated a statistically significant lower procedure duration for DGE compared to BAGE (35 min for DGE vs. 90 min BAGE). The unassisted DGE method is expected to have a lower procedure duration, as fewer materials and steps are necessary. The results from our review (49 min for DGE and 66 min for BTGE; 90 min BAGE vs. 55 min for EPASS) may have been influenced by outlier studies, such as Itoi et al. [12] (25 min) in EPASS. This presented procedure duration result differs from other studies of the same technique. Having developed the double-balloon catheter used to perform EPASS, the high level of expertise of Itoi et al. [12] in the technique could justify the better results they have achieved. The lack of statistical significance in this outcome may have been influenced by the low number of studies and patients for the BTGE techniques, as a 25% lower procedure duration could be clinically and, possibly, statistically relevant if a more significant pool of patients should be included.

To confirm their technical and clinical equivalence, we opted to compare BAGE and EPASS, the balloon-assisted techniques. Our analysis showed no statistical difference between the two in all the outcomes, except the rate of AEs. Combining these two techniques into just one category to compare with the more well-known DGE approach resulted in a systematic review with a larger patient pool.

This subgroup analysis should also be interpreted with caution. BAGE had an 8.9% rate of AEs and 4.2% of SAEs, while EPASS had 28.5% and 9.3%, respectively. Although the rate of AEs had a statistically significant difference, the low number of studies and the small pool of patients could have influenced the results. A larger pool of patients could deliver better data on these analyses.

Despite this being the first systematic review and meta-analysis evaluating the outcomes of the different EUS-GE techniques, this study is not exempt from limitations. Caution should be practiced when interpreting these results, as one significant limitation is the quality of the studies included. It consists mainly of retrospective case series. All studies were eligible to be included because of the very limited comparative studies. We opted to exclude other techniques, such as rendezvous and retrograde, as they only appeared in small case series. We also excluded studies that did not explicitly describe the technique used or did not separate the results from one another. 

The EUS-GE being a novel procedure without a gold standard explains the various techniques developed and the studies with a few included patients. In addition, the procedures are concentrated in large referral centers, which interferes with the generalizability of these findings, compelling the authors to produce multi-technique and multi-center studies to attain a considerable sample size. The report of AEs in the included studies from this review had different classifications; thus, the conversion to the ASGE lexicon classification for endoscopic AEs may represent a source of bias in our study. Despite these limitations, our review, the only meta-analysis on this theme, shows strengths in demonstrating a summary of the efficacy and safety of the main EUS-GE techniques. 

The different novel EUS-GE techniques demand a steep and laborious learning curve. The novelty may still interfere with the results in comparison to other modalities of therapeutic procedures for GOO, such as SGJ. Mastering any technique may improve results, with lower AEs and faster procedures [13, 25, 42]. All the methods evaluated in this study are comparable and can be performed without additional harm to the patient. Further well-designed randomized clinical studies are warranted to compare the different techniques.

## Conclusions

In summary, this systematic review and meta-analysis demonstrated that EUS-GE is a safe and effective treatment for the palliation of GOO. With the correct execution, any of the analyzed techniques may be used to palliate GOO with similar technical and clinical outcomes. Although DGE presented a statistically significant lower rate of AEs and LOHS, which can be inferred as a safer procedure, the best approach should be individualized, considering personal and local expertise and availability of material and devices.
